# Contribution of Cerebellar Glutamatergic and GABAergic Systems in Premotor and Early Stages of Parkinson’s Disease

**DOI:** 10.3390/ijms262110754

**Published:** 2025-11-05

**Authors:** Clelia Pellicano, Daniela Vecchio, Federico Giove, Lucia Macchiusi, Marco Clemenzi, Claudia Marzi, Mariana Fernandes, Flavia Cirillo, Silvia Maio, Claudio Liguori, Fabrizio Piras, Federica Piras

**Affiliations:** 1 Neuropsychiatry Laboratory, Clinical Neuroscience and Neurorehabilitation Department, IRCCS Santa Lucia Foundation, Via Ardeatina 306, 00179 Rome, Italy; c.pellicano@hsantalucia.it (C.P.); l.macchiusi@hsantalucia.it (L.M.); f.piras@hsantalucia.it (F.P.); federica.piras@hsantalucia.it (F.P.); 2 Neuroimaging Laboratory, IRCCS Santa Lucia Foundation, Via Ardeatina 306, 00179 Rome, Italy; f.giove@hsantalucia.it (F.G.); m.clemenzi@hsantalucia.it (M.C.); c.marzi@hsantalucia.it (C.M.); 3 Centro Ricerche Enrico Fermi, Via Panisperna 89a, 00184 Rome, Italy; 4 Department of Systems Medicine, University of Rome Tor Vergata, Via Montpellier 4, 00133 Rome, Italy; fernandes.mlp@gmail.com (M.F.);; 5 Neurology Unit, University Hospital of Rome Tor Vergata, Viale Oxford 81, 00133 Rome, Italy; flaviacirillo29@gmail.com (F.C.);

**Keywords:** Parkinson’s disease, isolated or idiopathic REM sleep behaviour disorder, magnetic resonance spectroscopy, γ-aminobutyric acid (GABA), glutamate/glutamine (Glx) system, neuropsychiatric symptoms, neuropsychological performance

## Abstract

Parkinson’s disease (PD) is a multisystem disorder, with early changes extending beyond basal ganglia circuitries and involving non-dopaminergic pathways, including cerebellar networks. Whether cerebellar dysfunction reflects a compensatory mechanism or an intrinsic hallmark of disease progression remains unresolved. In this cross-sectional study, we examined how cerebellar γ-aminobutyric acid (GABA) and glutamate/glutamine (Glx) systems, as well as their excitatory/inhibitory (E/I) balance, are modulated along the disease course. As to ascertain how these mechanisms contribute to motor and non-motor features in the premotor and early stages of PD, 18 individuals with isolated REM sleep behavior disorder (iRBD), 20 de novo, drug-naïve PD (dnPD), and 18 matched healthy controls underwent clinical, cognitive, and neuropsychiatric assessments alongside cerebellar magnetic resonance spectroscopy (MRS, MEGA-PRESS, 3T). While cerebellar neurotransmitter levels did not differ significantly across groups, dnPD patients exhibited a shift toward hyperexcitability in the E/I ratio, without correlation to clinical or cognitive measures. In contrast, in iRBD, an inverse relationship between heightened GABAergic activity and neuropsychiatric symptoms emerged. These findings suggest an early, dynamic cerebellar involvement, potentially reflecting compensatory modulation of altered basal ganglia output. Our results support cerebellar GABA MRS as a promising biomarker and open perspectives for targeting non-dopaminergic pathways in PD.

## 1. Introduction

Extensive evidence highlights Parkinson’s disease (PD) as a multisystem disorder, with early involvement of non-dopaminergic networks beyond basal ganglia (BG) circuitries. Among these, alterations in cerebellar circuits have been implicated. The cerebellum and BG work together as part of a dynamic, interconnected network, exchanging information through direct subcortical pathways and shared thalamic targets [1]. The cerebellum, long recognized for its role in motor coordination, is increasingly understood to participate in cognitive and emotional processes, and its dysfunction is now considered a key contributor to both motor and non-motor symptoms (NMS) in PD [2]. It is likely that the cerebellum plays both pathological and compensatory roles in PD within the broader BG–cortex–cerebellum network. Recent studies show cerebellar abnormalities in PD patients, even at early stages. The largest and most comprehensive analysis of cerebellar volume to date, a worldwide collaboration to which we contributed, demonstrated region-specific alterations in anterior and posterior lobes associated with different clinical stages of PD, supporting early cerebellar involvement in the disease [3]. Functional neuroimaging studies demonstrated altered connectivity and hypermetabolism in the cerebellum, further confirming cerebellar contribution to motor and cognitive functions in PD [4,5,6].

Whether cerebellar dysfunction reflects a maladaptive compensatory mechanism or an intrinsic feature of disease progression remains a matter of ongoing investigation [7].

Beyond structural, functional, and metabolic changes, neurochemical alterations both within and outside the BG connections play a pivotal role in PD pathophysiology [8,9], especially considering the glutamate/glutamine (Glx) and the g-amino butyric acid (GABA) systems [10].

Glx and GABA neurotransmission regulate tonic inhibition and excitability within the central nervous system (CNS), playing a key role in maintaining the excitatory/inhibitory (E/I) balance essential for normal neural function [11]. This balance modulates input–output dynamics in cortical circuits, shapes responses to external stimuli, and supports efficient information processing and transmission [12]. Disruptions in E/I balance have been linked to the pathophysiology of several neuropsychiatric conditions, including schizophrenia [13] and autism [14]. Beyond their widespread roles in the CNS, these systems have specific importance in the modulation of BG and cerebellar circuits.

Both Glx and GABA can be probed noninvasively using magnetic resonance spectroscopy (MRS) [15], and MRS studies have definitively furthered our understanding of these neurochemical changes in PD [16]. Investigating cerebellar GABA levels and cognitive interference in PD, our group has shown that the relationship between cerebellar GABA-dependent tonic inhibition and response inhibition was reversed in PD patients (with respect to what was observed in healthy controls) [17]. We hypothesized that altered cerebellar–cerebral GABAergic neurotransmission may compensate for cognitive impairment linked to prefrontal dopaminergic dysfunction in PD, although increased cerebellar GABA may also reflect an early disease-related change [17]. However, as our findings came from medicated PD patients, dopamine therapy may have normalized cerebellar inhibition and cognition.

To date, there is a lack of studies examining the role of the cerebellar GABA and Glx systems in untreated, de novo drug-naïve PD patients (dnPD) patients and in those with idiopathic or isolated rapid eye movement (REM) sleep behavior disorder (iRBD), the strongest prodromal marker of α-synucleinopathies, especially PD and dementia with Lewy bodies (DLB) [18,19]. Longitudinal studies have shown that over 80% of individuals with iRBD eventually convert to a defined synucleinopathy, highlighting its value for early identification of at-risk populations [20,21]. Interestingly, alteration in the activity of GABAergic neurons in the brainstem is associated with the development of RBD in an animal model study [22].

This study aims to address these gaps by investigating how cerebellar GABA and Glx systems, as well as their E/I balance (Glx/GABA), are modulated in iRBD and dnPD patients and how this balance contributes to motor and NMS in the premotor and early stages of the disease.

We hypothesize that, given the importance of BG and cerebellum networks, alterations in the E/I balance emerge during the earliest stages of PD. By examining these early neurochemical alterations, we aim to offer insights into prodromal and very precocious compensatory mechanisms in PD, potentially advancing biomarker development for early intervention strategies.

## 2. Results

### 2.1. Clinical Evaluation and MRS Metabolites

The three groups did not differ in terms of age, gender distribution, or years of education (Table 1). As expected, iRBD and dnPD groups had different modified Unified Parkinson’s Disease Rating Scale [23] (MDS-UPDRS) part II and part III scores (two-tailed *t*-test for independent samples, unequal variances, *p* < 0.001), while they were homogeneous for the remaining clinical characteristics (Table 1).

A Shapiro–Wilk test showed that only the Glx_L and the Glx/GABA_R were not normally distributed in HC (respective significance for the Kolmogorov–Smirnov test, *p* = 0.031 and *p* = 0.041), while the other MRS variables were either normally distributed or had equal variance in each group. Therefore, parametric ANOVAs tested the potential effect of group (HC, iRBD, and dnPD) on normally distributed metabolite concentrations while a non-parametric test for three groups was employed to investigate differences in non-normally distributed variables. Table 2 reports metabolite concentrations and their ratio in the three groups. A Kruskal–Wallis test demonstrated a significant effect of group for the cerebellar Glx/GABA_R ratio (H = 7.216, df = 2, *p* = 0.027) (see Table 2).

Mann–Whitney tests for post hoc comparisons revealed that the cerebellar Glx/GABA_R ratio was greater in dnPD as compared to both HC (U = 86, *p* = 0.017) and iRBD (U = 93, *p* = 0.03) (Table 3). Such a result indicates the presence of a higher cerebellar Glx concentration in respect to GABA in dnPD, which suggests an imbalance toward excitation in this group. No further differences were found when comparing metabolite concentration among groups (Table 2).

No significant correlation was observable in either group between the MRS variable differentiating the groups and the MDS-UPDRS-III score.

### 2.2. Neuropsychiatric Evaluation and MRS Metabolites

Non-normally distributed variables (identified in Table 2 with #) were subject to non-parametric ANOVAs (Kruskal–Wallis test), demonstrating a significant effect of group for neuropsychiatric scores in terms of anxiety scores as measured by the Hamilton Anxiety Rating Scale, HARS [24] (H = 15.688, df = 2, *p* < 0.001), the severity of either somatic or total depressive symptoms assessed by the Beck Depression Inventory, BDI [25] (BDI-Som, H = 10.521, df = 2, *p* = 0.005; BDI-Tot, H = 8.192, df = 2, *p* = 0.017), as well as for apathy symptom severity (Apathy Scale—AS [26], H = 7.136, df = 2, *p* = 0.028) (Table 2). Specifically, Mann–Whitney tests for post hoc comparisons revealed that anxiety and depressive symptoms were significantly higher in both dnPD and iRBD as compared to HC, while apathy scores were abnormal in dnPD only (Table 3). No differences were detected in the Toronto Alexithymia Scale-20 item—TAS-20) [27] among groups. Non-parametric correlational analyses revealed that the Glx/GABA_R ratio was significantly negatively correlated with anxiety scores (*p* = 0.043) and both the somatic and the total depression symptoms severity scores (respectively, *p* = 0.007, *p* = 0.01) in the iRBD group, indicating that a lower ratio corresponded to a higher symptom severity. In the iRBD group, there was also a significant negative correlation with difficulties in identifying one’s own feelings (i.e., TAS-F1) (*p* = 0.032), with a higher ratio corresponding to a lower severity in such alexithymic symptoms (Table 4).

### 2.3. Neuropsychological Assessment and MRS Metabolites

Parametric and non-parametric ANOVAs (according to variables’ distribution; see Table 2 and Table 3, where # denotes non-normally distributed metrics) demonstrated a significant effect of group in neuropsychological performances in terms of verbal memory (Rey’s 15-word test, immediate and delayed recall [28], respectively, F= 9.304, df = 2;53, *p* < 0.001; Kruskal–Wallis H = 9.084, df = 2, *p* = 0.011) and cognitive flexibility/shifting abilities (as indexed by perseverative errors in the Modified Wisconsin Card Sorting Test short form (WCST-msf) [29,30]; Kruskal–Wallis H = 7.473, df = 2, *p* = 0.024) (Table 2). Mann–Whitney tests for post hoc comparisons revealed that both dnPD and iRBD (which did not differ one another) showed worse performance than the HC group (Table 3). Non-parametric correlational analyses revealed that different neuropsychological measures were significantly correlated with the Glx/GABA_R ratio, either in the iRBD or the HC group (Table 4). Specifically, constructional praxis (as measured through the immediate Copy of Rey–Osterrieth picture) [31,32]) and cognitive flexibility abilities significantly correlated (respectively, positively with *p* = 0.024 and negatively with *p* = 0.015) with the metabolite ratio in the iRBD group, indicating that the higher the ratio, the better the performance. A similar positive association among Glx/GABA_R levels and simple praxis abilities also resulted in the HC group (*p* = 0.015). Moreover, in the HC group, the metabolite ratio showed significant correlations with subjects’ verbal memory and information-processing-speed measures (respectively, positive with *p* = 0.038 and negative with *p* = 0.012), indicating that the higher the ratio, the better performance.

## 3. Discussion

The present work conducted a multi-level examination, including biochemical (glutamatergic/GABAergic), clinical, and behavioral levels, to investigate the potential etiological contribution of neurochemical changes in the cerebellum to the development of motor and NMS in the prodromal and very early stages of PD. Given that it is yet to be established whether changes in the cerebellar GABA and Glx systems and their balance are part of the widespread neurodegenerative process in PD, we intended to investigate if aberrant cerebellar GABAergic metabolism is observable in the very early stages of the disease (i.e., in dnPD patients) and whether it can be considered an etiological contributor to the development of the disorder (being therefore also discernable in its prodromal iRBD phase). De novo drug-naïve PD patients (dnPD) were also involved so as to rule out any potential normalization in the GABA/Glx system due to dopaminergic supplementation [17,33].

A first crucial finding of the present study is that neither iRBD nor early-stage PD differed in cerebellar neurotransmitters levels from healthy comparators (nor between the two populations). A significant difference was only observable for the Glx/GABA ratio in the right cerebellar hemisphere, wherein dnPD patients exhibited an augmented ratio compared to both iRBD and HC (which did not differ one another). This finding points to a state of neural hyperexcitability, thus suggesting dysfunction in the E/I balance of the cerebellum, where excitation overpowers inhibition, potentially leading to regional hyperactivity, disordered functional connectivity, and network instability. As a matter of fact, consistent metanalytic evidence [6] demonstrated either hypermetabolism or hyperactivity in the cerebellum of PD patients at rest and in response to cognitive and motor paradigms. The cerebellar hypermetabolism (covarying with metabolic reductions in frontal and parietal association areas) is assumed to be distinctive of the PD cognitive phenotype [5] while increased cerebellar activity may be a compensatory mechanism to cope with motor demands [6].

Although previous MRS investigations were not specifically focused on the cerebellum, it has been suggested that the pathophysiology of PD might be modulated by GABA, as motor symptoms may be caused by increased activity in the basal ganglia due to downregulation of GABAergic nigrostriatal projection neurons [33]. We speculate that alterations in the same pathway may also explain the observed reduced cortical GABAergic uptake (which was related to the clinical lateralization of symptoms in drug-naïve patients) [34], which is indicative of an imbalance between local GABA inhibition and dopamine excitation, as the first is no longer upregulated in response to increased dopamine release [34]. Indeed, downregulation of local GABAergic interneurons (as observed, for example, in the striatum) could be the first response to decreased dopaminergic modulation [10,35], leading to an E/I imbalance, with a resulting shift toward hyperexcitability. Enhanced excitability in the primary motor cortex (related to GABA concentrations), possibly interfering with normal processing, is associated with motor symptom severity, suggesting that GABA depletion may contribute to motor symptom expression [36]. Indeed, in neuropsychiatric conditions characterized by cerebello-thalamo-cortical circuitry abnormalities comparable to those observed in PD, reduced cortical inhibition (demonstrated by decreased motor-evoked potentials and altered intracortical inhibition) was mediated by changes in cerebellar GABA levels [37]. As cerebellar GABAergic inhibition concurs to refine cognitive and motor processes, inhibitory deficits contribute to increased neural noise, which, in turn, affect motor network connectivity, leading to slower motor responses [38]. This would suggest that a dysfunction in cerebellar inhibitory control, possibly due to failures in properly modulating the cerebello-thalamo-cortical circuit, may also contribute to the slowness and difficulty in movement initiation seen in bradykinesia [36].

Nevertheless, since the cerebellar involvement in PD remains largely unclear, especially in regard to the pathological and/or compensatory mechanisms at play, it is also possible that the reported cerebellar hyperactivation is indicative of the increased demands posed by movement impairments on certain cerebellar sub-regions [6]. However, no significant correlation was observed in our dnPD sample between the E/I balance in the right cerebellar hemisphere and the severity of motor symptoms as measured by the MDS-UPDRS-III, while metanalytic evidence reported a significant association between increased functional activity in the cerebellum and better motor clinical states [6]. However, the significant effect of group, and the absence of any other significant relationship with neuropsychiatric symptoms or cognitive performance in a population distinctively characterized by motor deficits, might at least suggest that the observed shift toward hyperexcitability concurs to motor symptom expression. The collateral finding that, in iRBD, in which by definition no manifest motor symptoms are detectable, cerebellar metabolites (or their balance) did not differ from HC, further reinforces the notion of a potential relationship between cerebellar activity and motor function in PD patients [6]. Indeed, in our previous study on medicated PD patients [17], a significant negative correlation was observed between mean cerebellar Glx levels and levodopa equivalents, suggesting that a pharmacologically induced normalization of excitatory output from the cerebellum significantly contributes to the effective management of motor symptoms.

The same imbalance in the cerebellar E/I ratio could be a key factor not just in motor function but also in cognitive symptoms, and metabolic patterns associated with motor and cognitive functioning in PD may be orthogonal, i.e., statistically independent [39]. As a matter of fact, we previously reported an inverse correlation between the Glx/GABA ratio in the right cerebellar hemisphere and the Stroop test interference effect [17], implying that hyperexcitability in the cerebellum may enhance the ability to filter irrelevant information. Earlier MRS studies indicate that PD patients with substantial gray matter atrophy in extra-visual regions exhibit reduced inhibitory GABA levels, which may be related to an increase in neuronal excitability to compensate for inadequate visual input, albeit resulting in visual hallucinations [40]. Consequently, in PD patients with prefronto-cerebellar circuit anomalies and executive control deficits, heightened cerebellar excitability may be necessary to optimize the filtering of irrelevant information, as an imbalance toward inhibition negatively impacts performance [17]. Alternatively, the altered E/I balance in the right cerebellar hemisphere may be purposeful to restore the reduced functional connectivity between the cerebellum and large-scale cortical networks frequently observed in PD [41,42]. Specifically, diminished cerebellar connectivity with the salience network (crucial for detecting and prioritizing relevant stimuli) correlates with cognitive deficits. Therefore, alterations in the cerebellar excitatory–inhibitory equilibrium may enhance inter-network dynamics for information processing and propagation [43].

However, these hypotheses are only speculative since in the present investigation, neither motor symptom severity nor cognitive performance were related to the abnormal E/I ratio in the right cerebellum in the dnPD group.

A second intriguing finding is that in iRBD significant correlations were observable between the E/I balance in the right cerebellar hemisphere and both neuropsychiatric symptoms and neurocognitive performance. Although it has been suggested that more research is needed to explore the role of neurotransmitters in this prodromal stage of α-synucleinopathies [35], MRS has been scarcely employed in iRBD (e.g., Ref. [44]), and current neurochemical evidence is essentially limited to dopamine transport imaging to investigate potential subclinical nigrostriatal dopaminergic dysfunction [45]. However, GABA signaling is implicated in the disorder [46] and research in animals suggests that disruptions in glycine and GABAergic neurotransmission may be a primary cause or contributing factor to the development of iRBD [47].

Pertaining to neuropsychiatric symptoms, we found significant differences among groups. Interestingly, we observed a continuum of anxiety symptoms, with dnPD patients presenting scores above the cut-off for mild anxiety and iRBD individuals exhibiting a more severe symptomatology compared to HC (although within the range of scores for no/minimal anxiety) but not to PD. These results are consistent with our previous study on the cognitive and neuropsychiatric profiles of iRBD [48] and align with growing evidence indicating that individuals with iRBD manifest early symptoms comparable to those subsequently seen in overt PD [49,50]. In contrast to our previous findings (where medicated PD patients did not show apathetic symptoms), we observed significant differences in apathy scores only between dnPD patients and HCs. However, although this difference reached statistical significance, apathy scores in dnPD patients remained well below the clinical cut-off. This pattern is consistent with evidence showing that, although apathy may be detectable at diagnosis, in the earliest stages it is generally mild and often transient, with prevalence and severity tending to increase as the disease progresses. The contrasting evidence may also be attributable to the heterogeneity across PD subtypes, whereby apathy appears more prevalent in non-tremor-dominant phenotypes and in patients with cognitive impairments [51]. No group differences were found in TAS scores.

With regard to the main focus of our study, we found a significant correlation among anxiety, depressive, and alexithymic symptoms and cerebellar metabolites. More specifically, our findings indicate that the cerebellar E/I balance is negatively correlated with these neuropsychiatric symptoms exclusively in patients with iRBD, with increased GABAergic tone associated with increased symptomatology. This observation is pathophysiologically relevant, as it contrasts with the typically anxiolytic and antidepressant effects of enhanced GABAergic activity. Indeed, it is well known that pharmacological agents that augment GABAergic transmission are effective in reducing abnormal fear and anxiety-related behaviors, as evidenced by both preclinical animal models and clinical human investigations [52]. Furthermore, it is well established that the cerebellum plays a pivotal role in modulating the activity of the cortical networks involved in emotional regulation [53,54], seamlessly dampening or strengthening their synchronized activity by balancing excitatory and inhibitory neurotransmission [43]. Heightened connectivity among the cerebellum and the salience (and particularly the amygdala, a central hub for emotional processing), the default-mode, and the central executive networks is demonstrated in both patients with anxiety disorders and non-clinical individuals exhibiting elevated state or trait anxiety [55]. Additionally, previous evidence in PD revealed a state of hyperconnectivity between the cerebellum and the limbic system [42], which was associated with increased depressive and anxiety symptoms [41]. Since functional connectivity between brain regions is neurochemically specific, it might be the case that the herein observed imbalance toward cerebellar hyperexcitation in dnPD may underlie the onset of mild-to-moderate neuropsychiatric symptoms, possibly consequent to disturbances in functional networks involved in top-down emotion regulation [42]. Under this framework, the inverse correlation we observed between the E/I balance and anxiety, depressive, and alexithymic symptoms in iRBD could indicate an early dysfunction of cerebellar inhibitory circuits, or alternatively a maladaptive reorganization, possibly reflecting an attempt to compensate for the emergence of these symptoms. Indeed, an increase in GABAergic activity can reduce the synchronization of regions within already deregulated emotion modulation networks [43,56], being sufficient to fully compensate the emergence of alexithymic symptoms (not observable in our iRBD sample), while providing only partial compensation for depressive and anxiety symptoms, thereby contributing to their relative persistence. We hypothesize that, as the disease progresses from its prodromal phase, such supposedly compensatory mechanisms may gradually decline or become entirely insufficient to counteract the altered output from the dysfunctional basal ganglia. Consequently, in patients with overt PD, the cerebellum may no longer effectively modulate these circuits, which could account for the absence of any observable correlation between the E/I balance and neuropsychiatric symptoms.

Regarding neuropsychological performance, iRBD patients significantly differed from HC (but not from PD patients) in both immediate and delayed verbal memory retrieval, as well as in their capacity to suppress a previously effective response during a task requiring cognitive adaptability, despite the fact that their performance was still within the normative range. Statistically significant correlations were observable between the right cerebellar E/I balance and performance in a visuoconstructive task, with a shift toward hyperexcitability related to better performance, and between the same ratio and perseverative errors, with an imbalance toward inhibition decreasing the ability to change strategies in response to new information. These observations are partially in contrast with previous findings from our group [48] (where delayed retrieval of verbal information differentiated iRBD from PD patients with and without RBD) and with a plethora of studies demonstrating visuoconstructive, learning, and executive impairments in the disorder (e.g., Ref. [57]) as markers of a prodromal neurodegenerative state [58]. However, since the presence of cognitive deficits is a key predictor of a faster progression, iRBD with normal cognition (like in the present sample) may have a more benign disease course and a longer time to phenoconversion, or their cognitive functioning might be sustained by a higher cognitive reserve [48] or by peculiar brain morphological and metabolic patterns observable during the prodromal phase of iRBD [59]. As a matter of fact, the same relationship between a shift toward excitation in the right cerebellar hemisphere and better performance in a visuoconstructive task was observed in HC and iRBD, suggesting that the same mechanism is at play, whereby increased excitation in regions dedicated to particular cognitive functions positively correlates with performance [12]. As the cerebellum sustains the many cognitive demands of visuoconstructive tasks, from spatial processing to visuomotor coordination and executive skills like planning and error correction, the herein observed heightened neural excitation seems to be functional for maintaining performance within the normal range. Conversely, while no relationship was observed in HC among cerebellar metabolites (and their balance) and performance in a task requiring cognitive flexibility and the inhibition of no longer effective responses, imbalance toward inhibition was related to worse performance in iRBD. Although inhibitory mechanisms centered on GABA levels (and on the modulation of the E/I balance) support the human ability to suppress the repetition of information, leading to more efficient cognitive processing [60], the cerebellar-cortical interactions affecting the efficiency of transitioning between response strategies are, in individuals with typical neurological function, accompanied by a significant cerebellar activation [61]. Yet, as the observed shift toward excitation in the right cerebellum was related to increased perseverative responses, and considering the abnormalities of brain network dynamics already observable at rest in iRBD [62,63], it is possible that the heightened cerebellar activation was not sufficient to ensure efficiency in shifting between response alternatives. Anyhow, as switching abilities was within the parameters of normalcy, our finding would imply that, in iRBD with normal cognition, performance in executive tasks is still sustained by the same mechanisms observed in neurologically healthy subjects.

In sum, although a significant effect of group on cerebellar metabolites was observed only in the dnPD group, with an imbalance toward cerebellar hyperexcitability being possibly related to motor symptomatology, the cerebellar E/I balance was associated with neuropsychiatric and cognitive symptoms in iRBD only. Our molecular findings can be interpreted within a macro-circuit system framework, suggesting that cerebellar hyperexcitability in dnPD may reflect dysregulated GABAergic transmission affecting the cerebello-thalamo-cortical circuit and motor symptom expression, or may serve as a compensatory mechanism for increased motor demands. We posited that the inverse relationship observed in iRBD between elevated GABAergic cerebellar activity and neuropsychiatric symptoms might reflect early cerebellar inhibitory circuit dysfunction or an inadequate compensatory mechanism, potentially alleviating alexithymic symptoms while only partially relieving depressive and anxiety symptoms. Similarly, the correlation between increased cerebellar excitability and perseverative errors in cognitive flexibility tasks was analyzed through the physiological mechanisms governing transitions between response alternatives, representing a successful attempt to sustain performance within normative levels.

Before some concluding remarks, a few limitations of the present investigation must be acknowledged. First, it might be argued that since is not possible to distinguish with MRS the multiple sources of glutamate/GABA (as it cannot discriminate between synaptic and intracellular stores of metabolites), the relevance of MRS measurements in human neuroscience is questionable [11]. However, several lines of evidence demonstrated the high regional stability and specificity of Glx/GABA measurements, and numerous studies reported stable relationships between Glx/GABA levels and performance in tasks that are thought to depend on inhibitory or excitatory neurotransmission in certain brain areas [11]. As the modulatory role of the cerebellum in the psychophysical performance and neuropsychiatric variables considered herein has been robustly established, we strongly assert that these measurements are relevant for the cognitive/affective processes investigated here. Moving forward, given that initial neurochemical alterations within the cerebellum (particularly concerning the Glx/GABA systems and their balance) are increasingly acknowledged as integral components of the extensive neurodegenerative continuum in PD, we posit that MRS evaluation in the early stages of PD and its prodromal phase should be further utilized to potentially elucidate the pathogenesis of the disorder and its longitudinal progression.

A further potential limitation of the present study might be the small sample size, since it was determined assuming a large effect in order to potentially detect only differences that could be of clinical significance (given the relevance of the scientific question, since we intended to investigate the earliest neurochemical alterations potentially observable in PD). However, a post hoc power analysis on the difference between the E/I cerebellar balance in dnPD and that measured in HC and iRBD demonstrated that the analysis had 91% and 90% probabilities of correctly detecting a true effect (1 − β = 0.91 for the post hoc comparison between dnPD and HC, and 1 − β = 0.906 for the comparison between dnPD and iRBD). Equally, the analysis probing potential correlations among cerebellar metabolites and neuropsychiatric/neuropsychological measures was not underpowered, as the achieved power for the significant relationships observed in iRBD ranged from 0.71 to 0.90 (with a probability of failing to detect a significant association spanning from 29% to 9%).

Additionally, it should be acknowledged that the potential presence of RBD in the dnPD sample was explored only through a clinical interview (thus no instrumental evaluation nor validated questionnaire was used), implying that the accuracy of the resulting information is debatable. Despite the lack of a definite diagnosis, 60% of our dnPD patients had a strong clinical history of dream enactment, potentially casting doubt on the specificity of our results. However, had our findings been solely influenced by the presence of RBD, they would not exhibit orthogonality, as a discernible shift towards hyperexcitation in the right cerebellum was exclusively documented in dnPD (which was interpreted as related to the emergence of motor symptoms), whereas substantial correlations with neuropsychiatric/neuropsychological metrics were identifiable solely in iRBD. Indeed, we assume that although some clinical features may overlap in the two populations (like the presence of RBD), the documented findings elucidate the longitudinal trajectory of the disease, wherein compensatory neurochemical mechanisms are discernible and functional during the prodromal phase yet become ineffective or no longer operational as the disease advances.

Finally, we are aware that although they were unmedicated, the fact that a small proportion of participants was under psychotropic drugs may constitute an additional limitation. Since only a small number of participants were taking benzodiazepines or antidepressants, no subgroup analyses could be conducted. Acute or recent benzodiazepines use was controlled by instructing participants to abstain prior to scanning, minimizing potential acute effects. While benzodiazepines can modulate GABA levels, their impact on MRS-derived GABA remains debated, as MRS measures total GABA concentration rather than synaptic activity or receptor binding [64]. Similarly, the effects of SSRIs, SNRIs, and other antidepressants on MRS-measured GABA are highly variable, with studies generally underpowered and showing inconsistent findings across brain regions and populations [65]. Taken together, the low prevalence of medication use and the control of acute benzodiazepines intake suggest that these factors likely had minimal or no influence on our results.

## 4. Materials and Methods

### 4.1. Participants

This cross-sectional study included 20 persons with iRBD and 25 dopaminergic-naïve persons with PD (denovo, dnPD) patients. They were evaluated for inclusion at the Parkinson’s outpatient clinic of the IRCCS Santa Lucia Foundation of Rome and at the Neurology Unit, University of Rome “Tor Vergata”, Italy. According to international guidelines, diagnosis was made based on the UK Parkinson’s Disease Society Brain Bank diagnostic criteria for PD [66] or the International Classification of Sleep Disorders-3rd Edition (ICSD-3) criteria for RBD [67] (see below for specific inclusion criteria). The study also enrolled a sample of 20 healthy controls (HC) subjects, who were closely matched for age (± 2 years) and gender to the other patient samples.

Inclusion criteria for all participants were (1) age between 50 and 85 years, (2) at least eight years of education, (3) vision and hearing sufficient for compliance with testing procedures and (4) suitability for Magnetic Resonance Imaging (MRI) scanning. Specific inclusion criteria for iRBD were (1) compliance for video-polysomnography (v-PSG) examination; (2) no sleep-related hypoventilation, pulmonary insufficiency, or apnea-hypopnea or oxygen desaturation index ≥ 15/h at v-PSG; (3) no clinical diagnosis of Parkinson’s disease or any additional neurological conditions, as determined by an assessment conducted by an expert neurologist; (4) absence of iatrogenic causes of RBD; and (5) no signs of neurodegenerative diseases. Specific inclusion criteria for dnPD were (1) early stages of the illness (modified Hoehn and Yahr scale [68] ≤ 2) and (2) no exposure to any specific pharmacological intervention for PD (levodopa, dopamine receptor agonist, or monoaminoxidase inhibitors).

For all participants, common exclusion criteria were (1) presence of major medical illnesses (non-stabilized diabetes, obstructive pulmonary disease or asthma, hematologic and oncologic disorders, vitamin B12 or folate deficiency, pernicious anemia, clinically significant and unstable active gastrointestinal, renal, hepatic, endocrine or cardiovascular disorders, and recently treated hypothyroidism); (2) history of alcoholism or drug dependence and abuse; (3) head trauma; (4) any past or present major psychiatric and personality disorders, according to the DSM-5 criteria [69] (apart from mild-to-moderate mood or anxiety disorders); (5) Mild Neurocognitive Disorder as substantiated by a Mini Mental Examination State [70] score < 26 or diagnosis of dementia according to the Movement Disorder Society clinical diagnostic criteria [71]; (6) presence of severe artifacts on T1-weighted MRI images that degraded the quality of images as established through a visual check of images performed or poor Quality Assurance (QA) for MRS (see below, neuroimaging and MRS acquisition and processing); and (7) presence of any potential brain abnormality and microvascular lesion as apparent on conventional T2-weighted or fluid attenuated inversion recovery (FLAIR) scans.

Out of the original samples of participants, 5 were diagnosed with dnPD, 2 were diagnosed with RBD, and 2 HC were excluded due to (1) not completing the entire magnetic resonance exam (N = 3); (2) presence of MRI artifacts or brain abnormalities (N = 2); or (3) poor QA for MRS scans (N = 4). Consequently, the final study sample included 20 dnPD patients, 18 iRBD patients, and 18 HCs. Within each group, the number (and %) of subjects receiving antidepressant and/or benzodiazepines therapy was calculated (see Table 1). Participants taking benzodiazepines were instructed to avoid the medication within 24–36 h prior to scanning to minimize possible acute effects on GABA measurements.

The study was conducted in accordance with the Declaration of Helsinki, and the protocols were approved by the Ethics Committee of IRCCS Fondazione Santa Lucia Prot. CE/PROG.701 approved on 26 July 2018 for dnPD, and Prot. CE/2022_042 approved on 21 December 2022 for iRBD.

Each subject signed an informed consent form prior to enrollment as a waiver for study participation and for the handling of their personal data.

### 4.2. Subjects’ Assessment

#### 4.2.1. Clinical Evaluation

During enrollment, demographic and clinical information (i.e., age of onset, age of diagnosis, and pharmacological treatment) were collected from all participants.

Neurological anamnesis was performed by trained neurologists (CP and CL) with expertise on parkinsonism. Disease stage for both iRBD and dnPD patients was established based on the modified Hoehn and Yahr [68] scale (H&Y), and the severity of motor/non-motor symptoms was rated using the modified Unified Parkinson’s Disease Rating Scale [23] (MDS-UPDRS) and the NMS Scale [72] (NMSS).

#### 4.2.2. Neuropsychiatric Evaluation

Patients diagnosed with dnPD and RBD underwent a detailed neuropsychiatric evaluation carried out through standardized scales assessing apathy (AS) [26], anxiety (HARS) [24], depressive (BDI) [73], and alexithymia (TAS) symptom severity. The TAS-20 comprises three subscales assessing different facets of alexithymia: F1, difficulty in identifying feelings; F2, difficulty in describing feelings; and F3, an externally oriented analytic mode of thinking. The presence of Impulse Control Disorders (ICDs) was also evaluated using the questionnaire for impulsive–compulsive disorders in PD [74].

All groups were also screened for a current or lifetime history of DSM-5 mental and personality disorders using both the SCID-5-RV [75] and the SCID-5-PD [76].

#### 4.2.3. Neuropsychological Assessment

All participants underwent a comprehensive neuropsychological assessment, including the Mental Deterioration Battery [28] (MDB) and other standardized cognitive tests, at least one week after/before their MRS scans. Specifically, patients were tested for general cognitive functioning (Mini-Mental State Examination [70]—MMSE) and different cognitive domains, such as (i) verbal memory (MDB Rey’s 15-word Immediate Recall and Delayed Recall); (ii) language (MDB Semantic and Phonological Verbal Fluency); (iii) long-term visual memory (Delayed Recall of Rey–Osterrieth picture) [31,32]; (iv) complex constructional praxis (Copy of Rey–Osterrieth picture) [31,32]; and (v) executive abilities, such as attention shifting, attentional control, and response inhibition (Trail Making Test A/B [77]- TMT A/B, the WCST-msf, and the short version of the Stroop Word-Color Test [30]—SWCT). As in our previous work [17], and according to Italian normative data [30], response inhibition abilities were evaluated from the SWCT by computing a time interference effect (based on execution time) and an error interference effect (based on number of errors).

### 4.3. Neuroimaging Procedure

#### 4.3.1. MRI and MRS Acquisition

MRI and MRS were acquired on a 3T Siemens Prisma scanner (Siemens Medical Solutions, Erlangen, Germany) equipped with high power shim amplifiers using a 64-channel receive head-and-neck coil and a 2-channel transmit body coil. Head position was fixed with foam padding to minimize movements.

T1-weighted (T1w) structural magnetic resonance images (TR = 2200 ms, TE = 2.2 ms, matrix = 288 × 288, FOV = 256 × 256 mm^2^, slice thickness = 0.9 mm, voxel size = 0.9 × 0.9 × 0.9 mm^3^, flip angle = 8°, TI = 900) were acquired for spectroscopic voxel placement and to normalize metabolite quantification upon subjects’ anatomy.

MEGA-PRESS data (TR/TE = 1500/68 ms, 51 Hz bandwidth editing pulse applied alternatively at 1.9 ppm and 7.5 ppm) were acquired in two voxels on the left and right cerebellum. The sequence included VAPOR (VAriable Power Optimized Relaxation delays) water suppression (Bandwidth 65 Hz) [78] and Outer Volume Saturation (OVS).

Before spectroscopy, first and second order shims were automatically adjusted using FASTMAP [79]. Transmission power, VAPOR reference flip angle, and the last VAPOR delay before the last two OVS blocks (T7 [80]) were optimized subject by subject with appropriate scanning cycles.

For each site, four spectra were acquired (2 × 32 transients each). Reference frequency was set at 3 p.p.m. and re-set before the acquisition of each spectrum to compensate for frequency drift. Transients were saved separately for further processing. Unsuppressed water signal was acquired in the same voxel for referencing and eddy current correction with the same parameters, but TR = 5000 ms.

**Figure 1:**
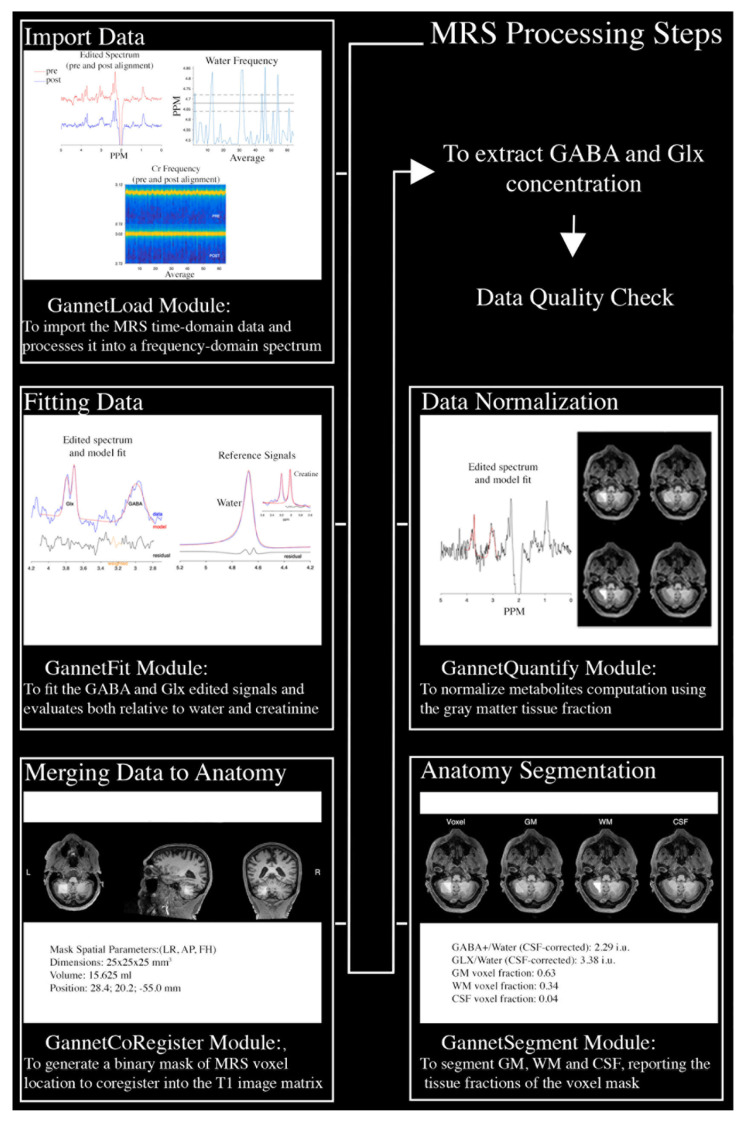
Schematic depiction of MRS data processing steps and employed Gannet 3.1 modules for data processing.

The MRS acquisition voxels (size 2.5 × 2.5 × 2.5 cm^3^) were positioned, taking care to minimize signals coming from both cerebrovascular fluid (CSF) and skull, and the voxel size was sufficient to include each cerebellar hemisphere (see Figure 1). T2-weighted and Fluid-Attenuated Inversion Recovery (FLAIR) sequences were acquired to clinically screen for possible brain pathology.

#### 4.3.2. MRS Processing

Each acquisition was processed individually with Gannet 3.1 [81] (open-source software downloadable at www.gabamrs.com/gannet, accessed on 6 October 2025), a Matlab-based quantitative analysis tool, specifically developed for GABA MEGA-PRESS spectra. For this purpose, GannetLoad, GannetFit, GannetCoRegister, GannetSegment and GannetQuantify modules were used for the MRS processing. Specifically, MRS time-domain data were imported, eddy current was corrected using the unsuppressed water data, lines were broadened by 2 Hz. Individual spectra were phased and frequency aligned using Spectral Registration [82] and summed in edited and non-edited data. The difference spectrum was then fitted and GABA and Glx quantified relative to water, using a Gaussian-Lorentzian model from the unsuppressed water spectrum.

A binary mask of the voxel location in the T1w image matrix was obtained with GannetCoregister. An SPM-based segmentation of the T1w anatomical image was performed, and the fraction of GM, WM, and CSF within the voxel binary mask was computed. Finally, metabolite concentrations were corrected for partial volume effects by assuming that they were present only in the GM fraction.

**Figure 2:**
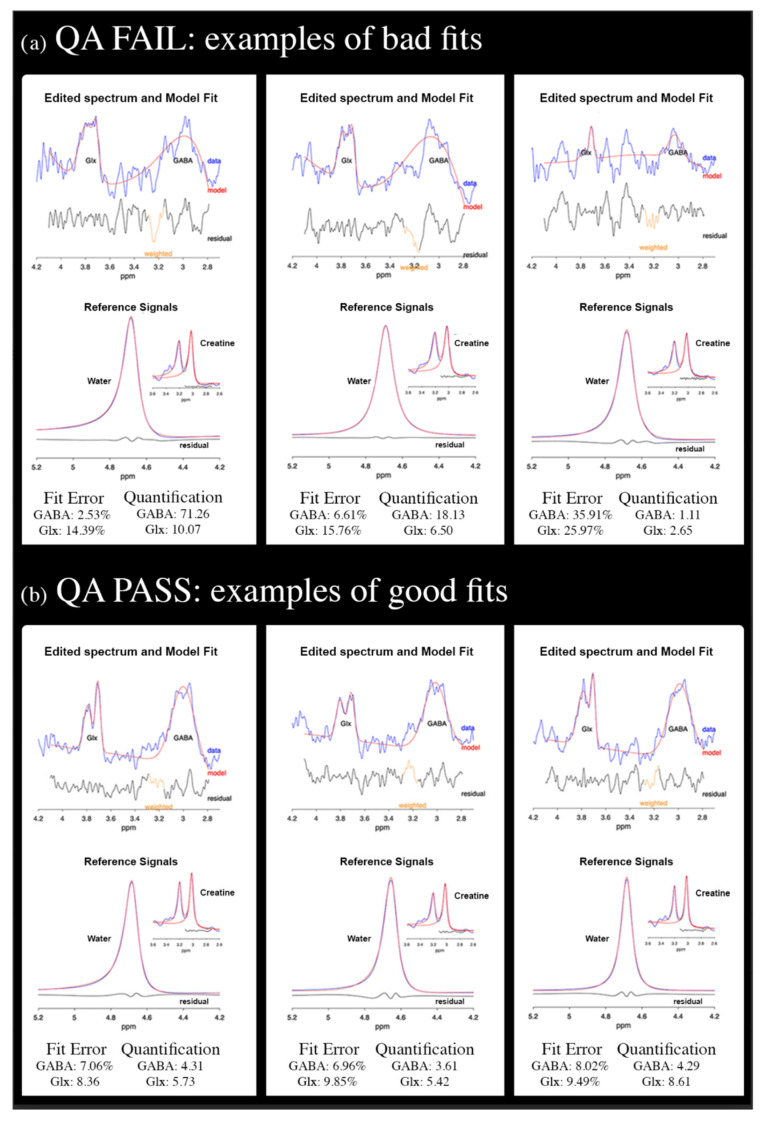
(**a**) Examples of bad fits not passing the quality check; (**b**) examples of good fits.

Data underwent MRS QA by excluding scans with either GABA or Glx fit errors greater than 15% or implausible fit values for both GABA/Water (i.e., <2.5 or >5.5) and Glx/Water ratios (i.e., <0 or >11) or poor quality of GABA or Glx fits (see Figure 2 for examples of good/poor QA fits).

A total of 9 MRS variables were computed from the described MRS processing. Specifically, after averaging of the 4 hemispheric cerebellar acquisitions, left and right concentrations of GABA and Glx metabolites were extracted (GABA_L, GABA_R, Glx_L, and Glx_R, respectively). In addition, both left and right Glx/GABA ratios were calculated to investigate the individual biological excitation/inhibition balance (Glx/GABA_L and Glx/GABA_R). Finally, total GABA, Glx, and Glx/GABA concentrations (GABAavg, Glxavg, Glx/GABAavg) were computed for each subject as the mean of the left and right measurements.

### 4.4. Statistical Analyses

Demographic and clinical characteristics were compared among groups using one-way ANOVA or independent sample *t*-test for continuous variables, while chi-square tests were performed for categorical ones.

A priori power analysis was performed using G*Power (v. 3.1.9.6), using an alpha of 0.05, a power of 0.80, and a large effect size (Cohen’s f = 0.4), in order to determine the sample size required to detect effects that would have clinical significance. The result showed that such an effect size could be detected by an ANOVA with a minimum number of 51 participants (≈17 per group).

The 9 MRS variables, as well as clinical and neuropsychological measures, were checked for normality distribution and homoscedasticity (respectively, with Shapiro–Wilk and Levene’s test) and then compared between group using ANOVA, or Kruskal–Wallis test, where appropriate. Post hoc analyses were performed in case of a significant group effect and Bonferroni correction applied for normally distributed variables. MRS variables that were significantly different among groups were correlated with either subjects’ clinical, neuropsychiatric, or cognitive measures using Pearson or Spearman correlation analysis, based on results from normality tests.

Statistical analyses were performed on IBM SPSS Statistics 29 software, considering *p* < 0.05 as the statistical threshold for significance.

## 5. Conclusions and Future Directions

To date, no previous studies have investigated cerebellar metabolites in iRBD. Although a definite conclusion regarding the pathological/compensatory nature of the described mechanisms cannot be drawn due to the absence of a significant link between cerebellar metabolites and motor symptom severity in dnPD, our findings advance our understanding of cerebellar involvement in the prodromal and early stages of PD. Indeed, we discerned distinctive associations between cerebellar metabolites and behavioral outcomes in the two populations, revealing significant correlations between the E/I balance and neuropsychiatric/neuropsychological metrics exclusively in iRBD and a shift toward hyperexcitability in dnPD which bore no relation to the onset of behavioral or motor symptoms. This pattern may reflect an initial attempt of the cerebellum to compensate for disrupted cortical regulation by modulating its E/I balance to counteract the altered output from the dysfunctional basal ganglia. As the disease progresses, however, this compensatory mechanism may progressively decline, ultimately failing to regulate these circuits, thus explaining the lack of associations in overt PD. Although further studies are needed to validate these findings, our results implicate non-dopaminergic cerebellar pathways in PD pathophysiology, extending the focus from the basal ganglia to additional neural structures. This expanded perspective not only highlights potential avenues for novel pharmacological interventions but also underscores the promise of cerebellar GABA MRS measurements as a diagnostic biomarker.

## Figures and Tables

**Table 1:** Demographic and clinical information for HC, iRBD, and dnPD.

	HC (N = 18)	iRBD (N = 18)	dnPD (N = 20)	t, chi^**2**^, F	df	*p*
Age, mean (sd)	67 (9.5)	68.6 (8.3)	67.1 (10)	**0.154**	2;53	0.858
Sex, male (%)	11 (61)	13 (72)	13 (65)	0.512	2	0.774
Years of education, mean (sd)	13.2 (3.8)	13.3 (4.3)	12.8 (4.7)	**0.065**	2;53	0.937
Age of onset, mean (sd)	-	65.3 (8.5)	65.7 (9.9)	*−0.14*	36	0.889
Age of diagnosis, mean (sd)	-	67.3 (8.5)	66.5 (10)	*0.258*	36	0.798
RBD illness duration, (years) mean (sd)		3.28 (2.1)		-	-	-
dnPD illness duration, (years) mean (sd)	-	-	1.45 (1)	-	-	-
Antidepressant, yes (%)	1 (5)	1 (5.6)	3 (15)	1.41	2	0.494
Benzodiazepines, yes (%)	1 (5)	0 (0)	3 (15)	3.32	2	0.191
MDS-UPDRS I, mean (sd)	-	5.78 (2.8)	6.6 (3.6)	*−0.779*	36	0.441
MDS-UPDRS II, mean (sd)	-	0.89 (1.4)	5.20 (3.2)	*−5.261*	36	<0.001 *
MDS-UPDRS III, mean (sd)	-	2.55 (2.3)	25.9 (9.4)	*−10.287*	36	<0.001 *
H&Y, median	-	-	2	-	-	-
Symptoms laterality, right (%)	-	-	11 (55)	-	-	-
ICDs, no (%)	-	18 (100)	19 (95)	0.924	1	0.336
NMSS, mean (sd)	-	28.3 (20.4)	33.8 (23.1)	*−* *0* *.778*	36	0.442

HC, healthy controls; iRBD, idiopathic or isolated rapid eye movement (REM) sleep behavior disorder patients; dnPD, de novo drug-naïve PD patients; t, chi^2^, F statistics from t-, chi-squared, and ANOVA tests; *p*, *p*-value; df, degrees of freedom; SD, standard deviation; H&Y, modified Hoehn and Yahr scale; MDS-UPDRS, Unified Parkinson’s Disease Rating Scale; ICDs, Impulse Control Disorders, NMSS, Non-Motor Symptoms Scale. * denotes statistical significance. Statistics from ANOVAs are highlighted in bold, those from *t*-tests in italics, and chi-squared statics are underlined.

**Table 2:** Group effect for cerebellar metabolite concentration and neuropsychiatric and neuropsychological measures.

	HC (N = 18)	iRBD (N = 18)	dnPD (N = 20)	F, H	df	*p*
Metabolite concentrations						
GABA_L	3.71 (0.48)	3.79 (0.46)	3.53 (0.46)	**1.59**	2;52	0.214
GABA_R	3.95 (0.57)	4.07 (0.74)	3.95 (0.53)	**0.225**	2;50	0.799
GABA_avg_	3.87 (0.35)	3.96 (0.43)	3.76 (0.40)	**1.166**	2;49	0.322
Glx_L #	6.83 (0.75)	7.27 (1.12)	6.55 (1.27)	*3.446*	2	0.179
Glx_R	6.92 (1.23)	7.15 (1.16)	7.47 (1.36)	**0.886**	2;52	0.418
Glx_avg_	6.95 (0.86)	7.19 (0.95)	7.01 (1.13)	**0.248**	2;50	0.781
Glx/GABA_L	1.88 (0.28)	1.93 (0.29)	1.88 (0.36)	**0.174**	2;51	0.841
Glx/GABA_R #	1.76 (0.24)	1.77 (0.33)	1.95 (0.24)	*7.216*	2	0.027 *
Glx/GABA_avg_	1.81 (0.22)	1.82 (0.17)	1.89 (0.19)	**0.943**	2;48	0.396
Neuropsychiatric scores						
HARS #	2.78 (3.26)	7.83 (4.46)	8.15 (6.37)	*15.688*	2	<0.001 *
BDI-Som #	1.61 (1.33)	3.67 (2.2)	3.35 (2.13)	*10.521*	2	0.005 *
BDI-Tot #	3.44 (3.1)	7.78 (5.9)	7.20 (6.1)	*8.192*	2	0.017 *
Apathy Scale #	0.94 (1.21)	3.06 (4.80)	3.10 (2.81)	*7.136*	2	0.028 *
TAS-F1#	7.9 (1.02)	9.44 (3.76)	9.0 (2.86)	*1.276*	2	0.528
TAS-F2 #	8.67 (3.77)	8.72 (4.4)	9.15 (2.9)	*0.777*	2	0.678
TAS-F3 #	15.44 (4.74)	15.78 (4.73)	16.75 (3.53)	*2.436*	2	0.296
TAS Tot. #	32.11 (7.35)	34.56 (9.3)	34.70 (4.3)	*1.965*	2	0.374
Neuropsychological performance						
Rey’s 15w Immediate Recall	48.22 (7.67)	36.61 (8.08)	38.85 (9.77)	**9.304**	2;53	<0.001 *
Rey’s 15w Delayed Recall #	10.89 (2.63)	8 (3.05)	8.45 (2.93)	*9.084*	2	0.011 *
WCST-msf Pers. Err. #	0.44 (0.86)	1.78 (1.93)	1.65 (2.28)	*7.473*	2	0.024 *

HC, healthy controls; iRBD, idiopathic or isolated rapid eye movement (REM) sleep behavior disorder patients; dnPD, de novo drug-naïve PD patients; F, H statistics from ANOVAs and Kruskal–Wallis tests; *p*, *p*-value; df, degrees of freedom; SD, standard deviation; HARS, Hamilton Anxiety Rating Scale; BDI-Som, Somatic subscale of the Beck Depression Inventory; BDI-Tot, total score of the Beck Depression Inventory; 15w, 15-word; WCST-msf Pers. Err., perseverative errors of the Modified Wisconsin Card Sorting Test short form; #, variables not normally distributed; *, *p*-value statistically significant. Statistics from parametric ANOVAs are highlighted in bold, those from Kruskal–Wallis test are in italics.

**Table 3:** Post hoc results for the significant group effect on cerebellar metabolite concentrations, neuropsychiatric scores, and neuropsychological performance.

Post Hoc Comparisons									
	HC vs. iRBD			HC vs. dnPD			iRBD vs. dnPD		
	Mean Diff.	U, t	*p*	Mean Diff.	U, t	*p*	Mean Diff.	U, t	*p*
Metabolite concentrations									
Glx/GABA_R #	−0.01	135	0.744	−0.19	86	0.017 *	−0.18	93	0.03 *
Neuropsychiatric group differences									
HARS #	−5.05	52	<0.001 *	−5.05	66	<0.001 *	−0.320	165	0.67
BDI-Som #	−2.06	72	0.004 *	−2.06	89.5	0.007 *	0.320	160.5	0.564
BDI-Tot #	−4.34	79	0.008 *	−4.34	101	0.021 *	0.580	170	0.769
Apathy Scale #	−2.12	127	0.279	−2.12	85.5	0.004 *	−0.040	145	0.317
Neuropsychological groups differences									
Rey’s 15w Immediate Recall	11.61	**11.6**	<0.001 *	11.61	**9.37**	0.004 *	−2.240	**−2.24**	1 (Bonf.p)
Rey’s 15w Delayed Recall #	2.89	76.5	0.007 *	2.89	97.5	0.015 *	−0.450	159	0.553 (Bonf.p)
WCST-msf Pers. Err. #	−1.34	89	0.012 *	−1.34	108	0.021 *	0.130	168	0.716 (Bonf.p)

HC, healthy controls; iRBD, idiopathic or isolated rapid eye movement (REM) sleep behavior disorder patients; dnPD, de novo drug-naïve PD patients; U, t statistics from Mann–Whitney’s and Bonferroni corrected post hoc tests; *p*, *p*-value; df, degrees of freedom; Mean Diff., mean differences; U, Mann–Whitney score; HARS, Hamilton Anxiety Rating Scale; BDI-Som, Somatic subscale of the Beck Depression Inventory; BDI-Tot, total score of the Beck Depression Inventory; 15w, 15-word; WCST-msf Pers. Err., perseverative errors in the Modified Wisconsin Card Sorting Test short form; #, variables not normally distributed; *, *p*-value statistically significant; Bonf p, *p*-value adjusted for Bonferroni’s multiple comparisons correction. Post hoc statistics for the only normally distributed variable (Rey’s 15w Immediate Recall) are highlighted in bold.

**Table 4:** Significant results from correlation analyses among the Glx/GABA_R ratios and neuropsychiatric and neuropsychological measures.

Correlation Analyses				
Glx/GABA_R~Neuropsychiatric Measures				
		Spearman’s Coefficient	R^**2**^	*p*
iRBD	HARS #	−0.50	0.25	0.043
	BDI-Som #	−0.63	0.40	0.007
	BDI-Tot #	−0.60	0.36	0.010
	TAS-F1 #	−0.52	0.27	0.032
dnPD	No significant correlations			
HC	No significant correlations			
Glx/GABA_R~Neuropsychological Measures				
iRBD	Copy of R-O picture	0.54	0.29	0.024
	WCST-msf Pers. Err	−0.58	0.34	0.015
dnPD	No significant correlations			
HC	Rey’s 15w Delayed Recall	0.51	0.26	0.038
	Copy of R-O picture	0.58	0.34	0.015
	SWCT-Color, time	−0.593	0.35	0.012

HC, healthy controls; iRBD, idiopathic or isolated rapid eye movement (REM) sleep behavior disorder patients; dnPD, de novo drug-naïve PD patients; R^2^, coefficient of determination; *p*, *p*-value; #, variables not normally distributed; HARS, Hamilton Anxiety Rating Scale; BDI-Som, somatic subscale in the Beck Depression Inventory; BDI-Tot, total score for the Beck Depression Inventory; TAS-F1, Factor 1 of the Toronto Alexithymia Scale-20 item; 15w, 15-word; R-O Figure, Copy, Copy of Rey–Osterrieth picture; WCST-msf Pers. Err., perseverative errors in the Modified Wisconsin Card Sorting Test short form; SWCT-Color, time, time to complete the color denomination table in the Stroop Word-Color Test.

## Data Availability

Open data sharing is restricted by the terms of participant informed consent and ethical constraints, making the data available only upon reasonable request to the corresponding author.
